# Adaptive Behavior Change in Autism: Outcomes from a Comprehensive, Interdisciplinary Clinical Care Cohort

**DOI:** 10.3390/jpm16050242

**Published:** 2026-04-30

**Authors:** Kelly Olvany, Annie Aitken, Elysa J. Marco, Neil Hattangadi, Kevin A. Shapiro

**Affiliations:** 1 Cortica Healthcare, San Diego, CA 92121, USA; aba396@nyu.edu (A.A.); emarco@corticacare.com (E.J.M.); nhattangadi@corticacare.com (N.H.); kshapiro@corticacare.com (K.A.S.); 2 Department of Applied Psychology, New York University, New York, NY 10012, USA; 3 Neurological Institute, Children’s Hospital Los Angeles, Los Angeles, CA 90027, USA

**Keywords:** adaptive behaviors, autism, treatment models, growth curves

## Abstract

**Purpose:** The purpose of this study was to examine the effects of a medically centered, interdisciplinary treatment model on adaptive behavior in children with Autism Spectrum Disorder (ASD). The Cortica model involves a comprehensive program including behavioral and developmental therapies, overseen by a neurodevelopmental physician. Here, we investigated how adaptive behaviors change over time during care at Cortica. **Methods:** We analyzed changes in the Vineland Adaptive Behavior Scales over the course of Cortica care compared to a community sample comprising longitudinal data from the National Database for Autism Research (NDAR). **Results:** Using propensity score weights to match cohorts based on baseline functioning, multilevel growth curve models showed significant Cohort × Time interactions for the Adaptive Behavior Composite (ABC) score and all subscale scores, indicating increased growth in adaptive behavior skills for children in the Cortica cohort relative to NDAR. **Conclusions:** Results of this study highlight the importance of using adaptive behaviors as a primary outcome in clinical research studies and suggest that a personalized, multidisciplinary approach to intervention can result in improved adaptive behavior skills over time.

## 1. Background

The term Autism Spectrum Disorder (ASD) refers to a continuum of neurodevelopmental differences characterized in part by challenges in adaptive functioning, particularly in the domains of socialization and communication, but also more generally [1]. Assessment of adaptive functioning skills—or “adaptive behaviors”—is conducted routinely in clinical practice to identify the severity of challenges encountered by autistic individuals and to measure progress over time [2]. However, despite the clinical significance of adaptive behaviors, relatively little is known about the impact of specific medically based interventions on adaptive behavior skills in autism.

In general, adaptive behaviors reflect the global ability to operate functionally and independently in a particular environment [3]. Categories of adaptive behaviors include those related to an individual’s ability to receive and understand communicatory cues from others, self-sufficiently tend to personal health and hygiene, and maintain interpersonal relationships [1]. Challenges with these skills in a predominantly neurotypical environment are common in autistic individuals and are predictive of functional outcomes throughout the lifespan [4]. At the same time, compared to more fixed traits such as intelligence, adaptive behaviors are more malleable and sensitive to change in autistic populations [5,6], suggesting that focusing on these skills early in life may represent a particularly productive approach to the goal of increasing functional independence in adulthood [7].

The current standard-of-care for adaptive behavior challenges in ASD is Early Intensive Behavioral Intervention (EIBI), an umbrella category that includes Applied Behavior Analysis (ABA) and similar behavioral therapies. However, studies examining changes in adaptive behaviors among children receiving ABA have demonstrated decidedly mixed results [8,9]. The wide heterogeneity in response to treatment suggests that care based on ABA and similar methods on their own may not be sufficient to address the variable challenges faced by autistic individuals. Intensive behavioral therapies have several other drawbacks, including significant time and cost burdens [10]. By contrast, approaches that integrate ABA with developmental sensory and motor therapies—such as physical, occupational, music, and speech therapy—may show more promise for encouraging growth in adaptive behavior skills, especially when parent training is included [11]. Broadly speaking, interventions that incorporate behavioral or developmental techniques show benefits for adaptive behaviors compared to community models; however, effect sizes are generally small [8,12,13,14]. In the community, many (if not most) autistic children do receive therapies apart from EIBI, but these are, for the most part, regarded as treatments for “co-morbid” motor, sensory, and language deficits, rather than for adaptive behavior challenges related to autism.

We should note that pharmaceutical therapies are also frequently used to treat symptoms that may impair adaptive functioning in autistic individuals, although to date no medication has shown efficacy for core symptoms of ASD. As with behavioral therapies, meta-analyses of drug clinical trials have shown mixed effects, typically with only modest improvements in adaptive behavior measures [15,16]. Pharmaceutical and medical treatments for ASD have generally been studied without regard to concomitant behavioral and developmental interventions (and vice versa), in part because these treatments are usually prescribed and administered in different clinical settings. Heterogeneity in response to treatment for adaptive behavior challenges and other symptoms in ASD—whether the treatment is pharmaceutical, behavioral, or sensorimotor—is likely dependent on the interaction of biological, cognitive, developmental, and contextual factors [17]. It therefore stands to reason that an interdisciplinary, individualized approach integrating behavioral, developmental, and medical interventions may yield better results than a therapeutic program that does not take these various factors into account.

Cortica consists of an individualized combination of behavioral, developmental, and medical therapies overseen by a neurodevelopmental physician (neurologist or pediatrician). Although no two children in the Cortica cohort receive exactly the same intervention, the overall approach is interdisciplinary and standardized across care centers. The Cortica model shares some common features with an empirically supported intervention framework, ‘naturalistic developmental behavioral interventions’ (NDBIs). NDBI is an umbrella term used to describe therapies that integrate ABA principles with developmental science perspectives [18,19,20].

NDBIs are play-based, developmentally informed behavioral interventions that embed evidence-based teaching strategies into natural social interactions with the goal of accelerating social communication and learning in young autistic children. NDBI models share a set of core features, including teaching in everyday environments to promote generalization and spontaneous use of skills, with rewards that are intrinsically linked to behavior; child-initiated learning, with shared control of instruction between child and adult; the use of gradually faded prompts and shaping strategies; and a high density of learning opportunities with an emphasis on parent training [21,22,23,24].

The NDBI framework integrates Applied Behavior Analysis with developmental science, drawing on Piagetian and Vygotskian theories that emphasize the child’s active role in social learning. By targeting developmental precursors—such as joint attention and social imitation—within naturalistic play routines, this approach aims to accelerate functional gains beyond the trajectory of natural maturation [6]. The Cortica model implements these NDBI principles within a multimodal, physician-led framework, a strategy recently shown to improve adaptive behavior outcomes in longitudinal cohorts [2].

Cortica and NDBIs both emphasize the individuality of each child and the integration of several therapeutic approaches. However, in general, NDBIs are discrete therapeutic behavioral programs implemented independently of other interventions. While the Cortica model is also based in part on naturalistic principles informed by developmental science, and thus is aligned with the NDBI framework foundationally, Cortica extends beyond behavioral intervention to include medical evaluation and management as well as other developmental therapies that are typically delivered independently, including speech-language therapy, occupational therapy, and physical therapy.

Cortica is unique in that all therapeutic practices are overseen by a neurodevelopmental physician, and the framework is standardized across all Cortica sites. Under the Cortica model, physicians, therapists, and caregivers collaborate on treatment plans to harmonize medical and behavioral therapeutic approaches. Such a personalized form of intervention has obvious intuitive benefits from the perspective of caregivers, clinicians, and therapists, but implementing this kind of model requires a high degree of coordination, which is generally not supported by the existing infrastructure for healthcare reimbursement. For this reason, research is needed to examine whether this kind of unified, physician-directed model translates into improved adaptive behavior outcomes.

Further, while few studies have examined change in adaptive behaviors in response to treatment, even fewer have examined longitudinal change in adaptive behavior development over multiple timepoints in the course of clinical care. Though most existing studies investigating response to interventions in autism rely on two time points to infer change in symptoms, the development of adaptive behavior skills in children may vary over time; research using more than two time points can flexibly model changes in adaptive behavior over prolonged periods. Multilevel growth curve modeling techniques provide descriptive and individual-centered estimations of the patterns of behavioral change based on response to treatments [25].

The present study addresses this gap in knowledge by describing change in adaptive behaviors for children receiving care at clinical centers operated by Cortica. We aimed to examine change in adaptive behaviors over the course of care at Cortica, relative to change in children included in the National Database of Autism Research (NDAR; National Institutes of Health, Bethesda, MD, USA), who presumably received standard care for ASD in their communities. NDAR (now incorporated into the National Institute of Mental Health Data Archive, or NDA) is a repository for genomic and demographic data as well as clinical and behavioral assessments of individuals enrolled in multiple autism research studies since 2008 [26].

The types of data included in NDAR are highly heterogeneous, but include some assessments commonly used to measure outcomes in clinical settings. Our primary outcome measure was the Vineland Adaptive Behavior Scales (VABS; Pearson Assessments, Bloomington, MN, USA) [3,27], which has been shown to demonstrate good sensitivity to clinical improvement and thus represents a clinically useful marker of response to treatment [28]. The study was designed as a retrospective group comparison, leveraging propensity score weighting to improve causal inference between the two groups.

We hypothesized that children in both groups would show progress in adaptive behaviors over time, but that the rate of progress in adaptive behaviors for the Cortica sample would exceed that of the NDAR sample, based on the presumption of greater efficacy for a comprehensive, interdisciplinary care model compared to standard care. In other words, we assumed a priori that commonly utilized therapeutic approaches such as ABA, speech-language therapy, and occupational therapy are likely beneficial for the development of adaptive behaviors in autism. The intervention under study was not a specific therapy, but rather the coordination and personalization of multiple therapeutic modalities within a unified clinical framework.

In previous work, we and others have shown that socio-demographic variables can have a significant effect on trajectories of adaptive behavior outcomes in autism [2,29,30]. As a secondary aim, we therefore sought to determine whether socio-demographic variance affected progress in adaptive behaviors within the Cortica cohort. Comparable data were not available from the matched NDAR cohort, so it was not possible to control for socio-demographic variance in the between-groups comparisons.

## 2. Methods

### 2.1. Study Design

This was a retrospective group-comparison study using repeated measures. Parents completed questionnaires at the initial visit before treatment was started and on each subsequent visit throughout the course of treatment. In the Cortica cohort, data were collected post hoc from clinical records. In the “standard care” cohort, data were retrieved from the National Database for Autism Research (NDAR), a repository of the National Institute of Mental Health Data Archive (NDA). As noted above, NDAR is a resource for diverse, longitudinal and cross-sectional clinical and behavioral data from de-identified human subjects with autism and other neurodevelopmental disorders.

Generative AI (ChatGPT, OpenAI) was used to support this work in limited ways. Specifically, it assisted with editing sections of the manuscript for clarity, conciseness, and style, and provided suggestions for structuring the cover letter. In addition, it was used to check statistical analysis code and assist with the interpretation of results. All content was reviewed, verified, and approved by the authors, who take full responsibility for the accuracy and integrity of the manuscript.

### 2.2. Participants

We included all children with an ASD diagnosis between the ages of 19 and 90 months who had at least two assessments using the VABS in the Cortica research database between 2016 and 2022 (N = 807), as well as participants in NDAR for whom data from at least two VABS assessments were available (N = 103). The age range for the Cortica cohort was selected to match the NDAR cohort as closely as possible.

In the Cortica cohort, the diagnosis of ASD was made according to DSM-5 criteria, as assessed by a neurodevelopmental physician or nurse practitioner. This was confirmed with either the Autism Diagnostic Interview-Revised (ADI-R; Western Psychological Services, Torrance, CA, USA) [31] or the Autism Diagnostic Observation Schedule (ADOS; Western Psychological Services, Torrance, CA, USA) [32], performed by a clinician with specific training in these scales. Within the Cortica model, periodic assessment with the VABS is a standard component of ABA therapy; therefore, the sample included all children treated at Cortica who had received ABA long enough to have had two assessments and whose parents had consented to inclusion in the research database. The clinical acceptability of this intensive, interdisciplinary model is evidenced by the longitudinal retention of the cohort, as participants included in the analysis successfully maintained engagement across multiple therapeutic modalities between their baseline and follow-up assessments.

In the NDAR cohort, serial VABS scores were available for participants contributed by a longitudinal observational study of children with autism, non-autism developmental delay, or typical development led by researchers at the National Institute of Mental Health (Collection ID 2368, doi 10.15154/tzw6-qv81). We included only participants with a diagnosis of autism. All of these participants met DSM-IV-TR criteria for a diagnosis of ASD, as assessed by a clinician who met research reliability standards and confirmed with an ADI-R and an ADOS.

The study was conducted according to the principles outlined in the Declaration of Helsinki and was approved by the WIRB-Copernicus Group on 7 September 2022 (institutional review board protocol #20224562). We received a waiver of informed consent from the institutional review board, given that all aspects of this study were based on deidentified retrospective clinical data. Informed consent guidelines for NDAR are detailed in the Policy for NDAR (nih.gov).

Table 1 presents demographic information for children in each cohort. For the Cortica cohort, information about socioeconomic status, including caregiver income and education, was available for a subset of children based on questionnaires obtained at intake appointments; as socioeconomic status is known to influence outcomes in autism, we utilized this information in secondary analyses, to be described below. This information was not available for the NDAR cohort. Baseline demographic and clinical characteristics were compared between the Cortica and NDAR cohorts using independent samples *t*-tests for continuous variables and chi-square tests for categorical variables. Longitudinal changes in adaptive behavior were analyzed using Multilevel Growth Curve Modeling (MLM) to account for the nested structure of repeated measures within individuals, with model fit assessed via AIC and BIC, as detailed in Supplementary Table S2. The longitudinal dataset consisted of varying durations of care and assessment intervals. A detailed summary of the study period, the number of assessments per subject, and the baseline adaptive functioning levels (VABS ABC scores) is presented in Table 2.

### 2.3. Treatment

Cortica. Patients at Cortica with diagnoses of autism participate in a comprehensive, individualized therapeutic program that includes medical care and behavioral therapy (Applied Behavior Analysis, or ABA), as well as other developmental therapies (including occupational therapy, physical therapy, speech-language therapy, and music therapy) as clinically indicated. The program is overseen by a neurodevelopmental physician or nurse practitioner who is also responsible for optimizing pharmacologic and behavioral management of common associated medical conditions (such as epilepsy, anxiety, gastrointestinal symptoms, and sleep disturbance). The Cortica model aims to address medical, psychological, developmental, and behavioral concerns in parallel. Fidelity to this standardized framework was maintained through the multi-disciplinary oversight of a neurodevelopmental physician, who coordinated treatment plans across the various therapeutic modalities (e.g., ABA, speech-language therapy, occupational therapy, physical therapy, as outlined above) to ensure a unified clinical approach.

Because each child’s program is tailored to their clinical needs, any potential benefit attributable to the Cortica model at a macroscopic level is likely to relate not to any specific intervention but to the interdisciplinary nature of care. The care plan streamlines communication between physicians, therapists, and caregivers and therefore allows for collaborative problem solving to address medical and behavioral challenges. The program is insurance-based, and during the period under study all individual therapeutic interventions were reimbursed by insurance at standard rates. There was no additional charge to families for transdisciplinary care coordination.

**Figure 1:**
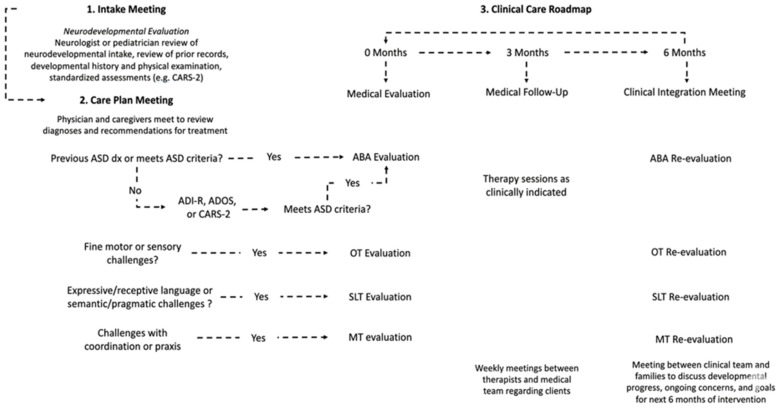
Cortica Clinical Care Roadmap and Intake Process. This flowchart outlines the three-stage progression from (1) the initial neurodevelopmental intake meeting and diagnostic evaluation to (2) the multidisciplinary care plan meeting, and finally (3) the six-month longitudinal clinical care roadmap involving medical follow-ups and therapy re-evaluations.

Figure 1 displays a schematic description of the Cortica framework. Families first attend an Intake Meeting, where neurologists or pediatricians review the patient’s neurodevelopmental intake information, prior medical records, developmental history and physical assessments, and any other prior standardized assessments. Following the intake meeting, families return to a Cortica clinic for their care plan meeting. Prior to March 2020, the care plan meeting was in person. However, after March 2020, the care plan meeting was transitioned to telehealth. During the care plan meeting, physicians and caregivers meet to review diagnoses and recommendations for treatment. Based on prior diagnoses or presenting challenges, patients are referred to appropriate ABA or developmental therapies. Thus begins the routine clinical care component of the Cortica model. At the first timepoint, medical, ABA, and appropriate developmental therapies are evaluated for the patient. At 3 months, therapy sessions are being conducted as clinically indicated, and patients are scheduled for a medical follow-up visit. During this time, weekly Rounds meetings are held between therapists and the medical team regarding clients’ progress. At 6 months, families return to Cortica for the clinical integration meeting, at which point the clinical team and families meet to discuss developmental progress, ongoing concerns, and the goals for next 6 months of intervention. More information on therapy dosage for the Cortica sample is reported in Table 3.

NDAR. As noted above, the NDAR cohort included participants recruited from the community for various autism research studies. Some data elements in NDAR do capture information about therapeutic intervention, but these were not available for the participants who also had longitudinal VABS scores.

Because all children in the NDAR cohort who were included in this analysis had been assigned a diagnosis of ASD, it is likely that their treatment consisted of therapies that were standard in the community at the time of their participation in autism research, including variable amounts of ABA, occupational therapy, speech-language therapy, and physical therapy. It is possible that some participants did not receive therapeutic interventions specific to autism. We therefore refer to the NDAR cohort as receiving “standard care,” as therapies for autism are generally not prescribed or delivered according to a unified clinical model. The NDAR group should be regarded as a natural history comparator rather than as a group exposed to a specific kind of therapy; in other words, changes within this group can be assumed to reflect the typical development of adaptive skills in children with autism.

## 3. Measures

Demographic Variables. Caregiver income and level of education were available for a subset of participants in the Cortica cohort. In a previous analysis, we demonstrated that socioeconomic variables are a significant predictor of outcome trajectories in autism, in line with earlier research [2]. Although comparable data were not available for the NDAR cohort, we included these variables in secondary analyses to assess potential biasing factors.

Adaptive Behavior. The primary outcome measure used was the Vineland Adaptive Behavior Scales (VABS) Parent/Caregiver Form [27]. The NDAR cohort utilized the second edition of the VABS [3], while the Cortica cohort utilized the third edition [27]. The parent-report VABS evaluates adaptive behaviors in five different domains: Social, Communication, Daily Living Skills, and Motor. Based on the summarized scores in each domain, an Adaptive Behavior Composite (ABC) is also calculated. Items are scored on a scale of 0 (never), 1 (sometimes), or 2 (usually) for the level at which the child can independently perform a skill. Each domain reflects a standard score based on a population mean of 100 and a standard deviation of 15. The Motor domain was not included in the current analysis, as the primary focus of the study was on social/communication behavior, independent daily living abilities, and overall adaptive behavior. The VABS was completed by the child’s primary caregiver (typically a parent); while the specific identity of the respondent (e.g., mother vs. father) was not consistently recorded as a discrete variable across both cohorts, all respondents were those with the greatest daily proximity to the child’s adaptive functioning. Potential implications of the differences between the second and third editions of the VABS will be discussed below in the Limitations section.

Analytic Plan. The goal of this analysis was to evaluate and compare rates of change in adaptive behaviors in two cohorts of children with autism: children receiving care at Cortica and those receiving typical care in the NDAR sample. To account for differences in severity of baseline adaptive behavior scores between the two cohorts, we conducted propensity score weighting on VABS ABC scores at the initial visit (T1). This approach allows inferences about rates of change between cohorts to be likelier attributable to differences in treatment received. Propensity score matching was conducted in the R environment using the MatchIt package (R Foundation for Statistical Computing; Vienna, Austria) [33]. Full matching on the propensity score was estimated using a probit regression of Cohort on Initial VABS ABC scores (see Supplementary Table S1 for full model parameters). This probit model was selected to effectively estimate propensity scores in a binary treatment context (Cortica vs. NDAR), allowing us to weight the samples and reduce the influence of baseline differences on our outcomes. A probit regression model was selected for propensity score estimation as it assumes a cumulative normal distribution of the latent variable, which is appropriate for the standardized distribution of baseline adaptive behavior scores. Full matching uses all units, so no participants were discarded after matching. Propensity score model estimation was evaluated by calculating balance statistics and eCDF diagnostics. To address concerns of exclusion bias introduced with propensity score sample weights, propensity score-matched samples were corroborated with a complete-sample analysis. Detailed parameters for the probit model and resulting balance statistics are provided in the Supplementary Information (Table S1).

To examine the effects of Cortica care relative to a standard care comparison group, we created multilevel longitudinal models with random intercepts and included the full matching weights in model estimations. Prior to fitting the full models, unconditional null models were estimated for each outcome to quantify the degree of non-independence in the data. ICCs ranged from 0.703 to 0.848 across the four VABS outcomes, confirming that the majority of variance in scores was attributable to between-person differences and justifying the use of multilevel modeling. Several multilevel models were created with each VABS score modeled as the dependent variable. At level one, or the time-variant level, we included Time and Age as independent variables. At level two, or time-invariant level, we included Cohort as an independent variable. All continuous independent variables were grand mean-centered. Finally, we included a cross-level interaction term of Cohort × Time to examine possible moderation in growth over time by Cohort. All significant interaction terms were probed with a simple slope analysis. Models were built sequentially, and fit was evaluated at each step. In all cases, the final interaction model demonstrated superior fit compared to null and predictor-only models based on AIC and BIC values (see Supplementary Table S2). This sequential modeling approach was utilized to ensure the highest level of statistical parsimony while accurately capturing the longitudinal complexities of real-world clinical data.

SES variables were unavailable for the NDAR cohort and were therefore not included in the primary between-group models. To address the possibility of cohort-level SES differences influencing group comparisons, we conducted robustness analyses within the Cortica cohort to examine whether income or education moderated VABS trajectories.

## 4. Results

### 4.1. Propensity Score Results

**Figure 2:**
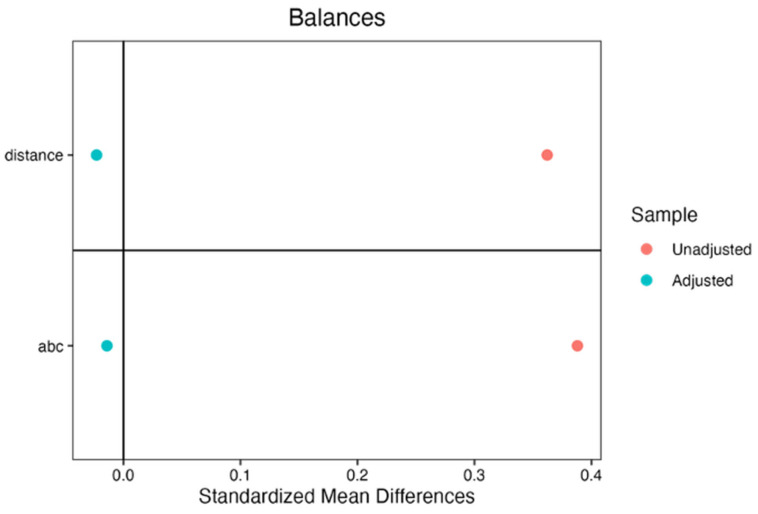
Covariate balance before and after propensity score weighting. Standardized mean differences for the distance (propensity score) and baseline ABC (Adaptive Behavior Composite) scores are shown for the unadjusted (red) and adjusted (blue) samples. The shift in the adjusted values toward zero indicates that the weighting procedure successfully balanced the Cortica and NDAR cohorts on baseline characteristics.

A probit regression model regressing cohort membership on baseline VABS ABC scores confirmed that baseline scores significantly predicted cohort membership (*b* = 0.019, SE = 0.003, z = 6.70, *p* < 0.001), supporting their use as the matching variable (see Supplementary Table S1). After matching, the standardized mean difference in initial VABS ABC scores between cohorts was substantially improved, decreasing from 0.388 pre-match to −0.014 post-match (see Figure 2), and eCDF was adequate (M = 0.004). Propensity-weighted multilevel model findings were corroborated by a complete-sample unweighted analysis (see Table 4).

### 4.2. Multilevel Models

Nested model comparisons using likelihood ratio tests indicated that model fit improved significantly at each step of the model-building sequence across all four VABS outcomes (see Supplementary Table S2). Adding main effects of Time, Cohort, and Age significantly improved fit over the null model in all cases (χ^2^ range: 23.44–41.31, all *p* < 0.001). The addition of the Cohort × Time interaction term further and significantly improved model fit across all outcomes (χ^2^ range: 28.95–65.02, all *p* < 0.001), supporting retention of the interaction term in the final models.

Results of propensity-weighted multilevel models are reported in Table 5. Results indicated a positive main effect of Time for all VABS outcomes; in other words, VABS scores increased over time, on average. There was a significant effect of Cohort on ABC, Socialization, and Daily Living Skills scores, with post hoc analyses indicating that the NDAR cohort had lower VABS scores on average in these domains relative to the Cortica cohort. Age was negatively associated with only the Socialization VABS domain, suggesting that older children have lower Socialization scores, on average.

**Figure 3:**
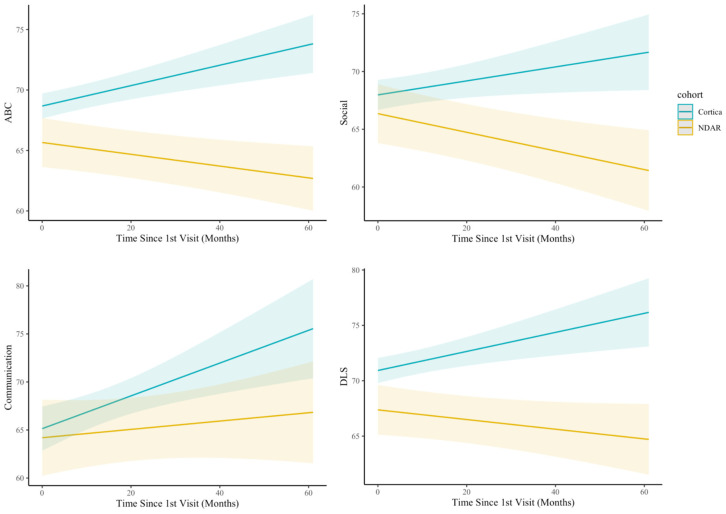
Longitudinal trajectories of Adaptive Behavior Composite (ABC) scores for the Cortica and NDAR cohorts. Data represent model-estimated slopes derived from linear mixed-effects modeling, which accounts for variable durations of enrollment (ranging from 3 months to 3 years) and varying intervals between measurements. These trajectories represent the predicted rate of developmental growth across the study period rather than raw time-point averages.

Our main research question pertained to the interaction term between Time and Cohort. As hypothesized, there was a significant Cohort × Time interaction for all VABS outcome scores. Simple slopes analysis of significant interactions is displayed in Figure 3. Analysis of simples slopes indicated that in the Cortica cohort, there was a positive association of Time with ABC (*b*_Cortica_ = 0.12, *p* < 0.001), Socialization (*b*_Cortica_ = 0.18, *p* < 0.001), Communication (*b*_Cortica_ = 0.08, *p* < 0.05), and Daily Living Score (*b*_Cortica_ = 0.11, *p* < 0.01) compared to the NDAR cohort, which demonstrated negative or null associations of Time with ABC (*b*_NDAR_ = −0.11, *p* < 0.001), Socialization (*b*_NDAR_ = −0.03, n.s.), Communication (*b*_NDAR_ = −0.21, *p* < 0.001), and Daily Living Score (*b*_NDAR_ = −0.11, *p* < 0.001). As shown in Table 5, these findings were supported by the non-weighted analysis.

### 4.3. Robustness Check Based on Socio-Demographic Information

To test any potential bias related to socio-demographic variables, which are known to impact outcomes in autism, we recreated multilevel models using the Cortica sample. We did not have comparable information regarding income and caregiver education levels for participants in the NDAR cohort, and therefore we were unable to control for socio-demographic variables in a direct comparison between the two groups.

First, we examined differences in VABS change over time for individuals who did and did not provide income or caregiver education level (a dummy variable was created for data provided or not provided). No significant interaction was present between time and the dummy variable (β < 0.02, n.s.), suggesting that there were no differences in outcome trajectories for individuals who did or did not have socio-demographic information in our dataset.

Second, we examined differences in VABS change over time based on income and education level for those individuals who did have socio-demographic information available. No significant interaction was present for the Time × Income or Time × Education Level (β < 0.01, n.s.). Thus, within the Cortica cohort, we did not find evidence that caregiver income or education had significant effects on VABS outcomes over time.

## 5. Discussion

Despite the clinical importance of adaptive skills as a target for therapies to ameliorate disability in children with neurodevelopmental differences, relatively little is known about the development of adaptive skills over time in this population. This study examined changes in adaptive behaviors over time in two cohorts of autistic children, one of which has previously been characterized in the literature [6].

A significant finding of this study was that children receiving care at Cortica showed greater improvement in adaptive skills over time compared to children from the NDAR database, who presumably received standard care for autism. Although NDAR did not capture information about therapeutic interventions for the participants we included with longitudinal VABS data, a study examining community utilization of interventions for a different subset of NDAR participants showed that the average intensity of therapy was 0.9 h per week for speech therapy, 0.8 h per week for occupational therapy, and 10.6 h per week for ABA or other forms of Early Intensive Behavioral Intervention [26]. In the Cortica cohort, the average intensity of each therapy was slightly lower (Table 3).

The finding that children in the Cortica cohort generally showed improvement in measures of adaptive behaviors, compared to the lack of improvement observed in the comparable NDAR cohort, was consistent across analytic methods, namely, propensity score weighted models and full-sample regression. To our knowledge, this is the first well-powered study to model gains in adaptive behaviors in a population for whom comprehensive data are available regarding the intensity and frequency of therapeutic interventions, as well as medical co-morbidities and management. Our findings in the control (NDAR) cohort are broadly similar to prior reports of changes in adaptive skills over time, which suggest limited improvement or deterioration for the majority of individuals [6,34]. This suggests that overall improvement in the Cortica cohort was not solely due to developmental maturation. A recent examination of the Cortica cohort over a longer timespan suggests that the gains in this group, relative to the standard care group, are likely to be maintained over time [2] though this group was not explicitly matched to the NDAR dataset.

One of the strengths of the current study was the use of propensity score weights, corroborated with a complete-sample analysis. The consistency of findings across both approaches demonstrates the robustness of these results to different analytic methods. The propensity-weighted method addresses the concern that the relative improvement in the Cortica sample could be attributed to less severe adaptive behavior deficits at the start of the treatment period. Across both analyses, there was a significant interaction between time and cohort, suggesting that the Cortica cohort showed greater gains in all domains of adaptive behaviors relative to the NDAR sample.

Contrary to our expectations, we did not show an effect of socio-demographic variables on outcomes in the Cortica cohort. In a separate study examining a larger sample from Cortica, we did find that socio-demographic variables were a significant predictor of outcome trajectories, in a model that also accounted for other clinical and demographic variables known at initiation of therapy. Our inability to detect an effect in this study may be due to the greater relative effects of other variables not controlled here, such as a history of developmental regression [2]. Notably, the absence of detected SES effects on change scores in this instance alleviates concerns regarding SES acting as a confounding variable between the NDAR and Cortica cohorts. The discrepancy with the Aitken (2025) [2] findings, where SES emerged as a top predictor, likely reflects methodological differences—specifically the use of a machine learning approach applied to latent class clusters within a broader variable set—rather than a true effect. Such predictors may not generalize across different analytic contexts.

Our results offer a preliminary suggestion that an interdisciplinary, medically based care model is superior to standard care in improving adaptive skills related to so-called core symptoms of autism, including difficulties with communication and social interaction. Effect sizes are similar to, or larger than, prior studies investigating the effects of specific behavioral or developmental therapies on adaptive skills in autistic children [13,14]. While the accelerated rate of skill acquisition in the Cortica cohort is statistically significant, further research is needed to determine the long-term maintenance of these gains and their impact on overall quality of life for families.

Broadly speaking, the primary goal of autism interventions is to reshape neural connections in order to support the development of adaptive behaviors and optimize psychosocial outcomes. Interventions that have been described in the clinical literature typically adopt a specific treatment approach that targets a well-defined set of behaviors in a single domain, such as speech, social responses, motor skills, or sensory processing [35,36,37,38]. However, these treatments do not take into account the interconnectedness and interdependence of abilities that are affected in autism. On the other hand, a therapeutic model that engages multiple cognitive domains simultaneously may provide synergistic benefits for the development of adaptive skills.

In addition, many autistic individuals are affected by physiologic symptoms including seizures, gastrointestinal disorders, and sleep disturbance, as well as neuropsychiatric symptoms of anxiety, inattention, hyperactivity, and motor tics, all of which can have a negative impact on neurodevelopmental outcomes [39]. The Cortica model adopts a multi-pronged approach that aims to address medical, psychological, developmental, and behavioral concerns in parallel. Our results suggest that an interdisciplinary “whole child” approach may indeed lead to better adaptive outcomes than standard, fragmented care.

## 6. Limitations and Future Directions

A notable limitation of the current study is the lack of a randomized control group. While this limitation was partially offset using propensity score weighting approaches, randomized control trials are the gold standard for inferring causal change. Unfortunately, in practice, it would be nearly impossible to randomize patients to standard vs. comprehensive care, as this is driven almost entirely by patient preference and access to services.

There were minor differences in inclusion criteria across cohorts: in the NDAR dataset, inclusion criteria were based on the DSM-IV-TR definition of autism confirmed by both the ADI-R and ADOS, whereas in the Cortica cohort, the DSM-5 definition was used along with only one confirmatory assessment. The versions of the VABS also differed between the two studies. While domain-level concordance between the second edition [3] and the third edition of the VABS [27] is generally high, especially for the ABC (concordance 0.85), the third edition tends to produce lower scores overall, particularly for individuals at lower levels of ability [40].

Changes in the DSM and VABS likely account, at least in part, for the difference in initial VABS scores across the two cohorts, and indeed the true differences in underlying adaptive skills may be larger than indicated by domain-level standard scores. It may also be the case that the Cortica cohort included some children whose symptoms fell short of the diagnostic threshold required for inclusion in the NDAR studies. However, propensity score weighting would again be expected to control for such variability in initial presentation. Moreover, baseline differences would not fully account for the key observation that outcome trajectories differed between groups—that is, the interaction between cohort and time.

The differences in clinical diagnostic criteria and assessment measures reflect the fact that data in the NDAR sample were collected over a period beginning roughly 10 years earlier than the Cortica dataset. During this time, it is possible there have also been changes in what generally constitutes standard care in autism. However, as we have already noted, Farmer [6] queried the NDAR database to investigate the utilization of therapeutic interventions prior to 2015. This analysis revealed roughly comparable average intensities of behavioral therapy, speech-language therapy, and occupational therapy to what we have reported for Cortica, suggesting that standards of care in the community have not shifted drastically. Although rates of autism screening and diagnosis have undoubtedly increased, this would not be expected to impact our analysis, as all children in both cohorts had confirmed diagnoses of autism. Furthermore, while the NDAR cohort provides a robust community benchmark, future research should utilize time-period-matched cohorts to control for historical shifts in standard-of-care practices and diagnostic criteria that may have evolved between the data collection windows of the two samples.

Despite the overall trend toward improvement in VABS scores in the current study population, there was still large individual variability in response to treatment. It is likely that contextual, biological, and developmental factors contribute to the heterogeneity in treatment efficacy demonstrated in various studies of interventions for autism (see also [2]). We note that the NDAR dataset did not include information about parental income or education or treatments received by children included in that cohort, so the effects of these variables could not be controlled or compared directly across cohorts. However, the availability of detailed information on socio-demographic characteristics, co-existing medical and psychological conditions, and intensity of specific therapeutic modalities within the Cortica cohort should allow us to examine the impact of each of these individual variables on outcomes in future analyses. On the other hand, NDAR does include other psychometric data, such as standardized intelligence testing, which were not available for the Cortica cohort. It will be helpful to gather such data for at least a subset of our population to assess the influence of cognitive factors not directly related to an autism diagnosis.

Future research should incorporate qualitative reports to more comprehensively evaluate patient and family satisfaction, tolerance, and perceived utility, providing a deeper understanding of the longitudinal impact of the Cortica model.

## 7. Conclusions

There is a critical need for evidence-based treatments to guide care planning for autistic individuals in ways that improve long-term outcomes, particularly functional independence. This is the first study of which we are aware that investigates the effects of an integrative, comprehensive, and personalized care model on multiple domains of adaptive behaviors, as compared with standard care. The results of this study provide preliminary evidence for the effectiveness of such a multidisciplinary approach for improving adaptive behaviors in autistic children.

## Figures and Tables

**Table 1:** Sample Descriptives.

	Cortica, N = 807	Standard Care, N = 103
Sex		
Female	156 (19%)	17 (17%)
Male	651 (81%)	86 (83%)
Age in months at initial timepoint of therapy	53 (42, 67)	47 (35, 59)
Income (primary caregiver)		
Less than $25,000	64 (20%)	
$25,000–$50,000	30 (9.1%)	
$50,000–$100,000	49 (15%)	
$100,000–$250,000	69 (21%)	
More than $250,000	13 (4.0%)	
Prefer not to answer	103 (31%)	
Unknown	479	103
Education level (primary caregiver)		
Less than High School	2 (0.6%)	
High School or GED	59 (18%)	
College Graduate	144 (45%)	
Post College Graduate	118 (37%)	
Unknown	484	103

Note. Age in months listed as median (IQR); all other variables represented as number of subjects (%).

**Table 2:** Study Diagnostic Measures.

	Cortica	Standard Care
VABS ABC score at initial timepoint	71 (12)	65 (9)
Interval between VABS (months)	6.5 (2.7)	12.4 (3.9)
VABS per subject	2, range 2–3	4, range 2–5
Duration of care (months)	12 (8)	23 (12)
Study period	2016–2022	2006–2014

Note. VABS ABC scores are reported as mean standard scores (standard deviation). Interval between VABS and duration of care are reported as the mean number of months (standard deviation). VABS per subject are reported as median and range. VABS: Vineland Adaptive Behavior Scales; ABC: Adaptive Behavior Composite.

**Table 3:** Characteristics of Therapies Received under the Cortica Model.

	Average Intensity of Therapy
Occupational therapy	0.77 (0.47)
Physical therapy	0.38 (0.32)
Speech-language therapy	0.61 (0.42)
Music therapy	0.65 (0.43)
Applied Behavior Analysis (ABA)	9.2 (5.3)

Note. Therapy intensity is reported in decimal hours per week (standard deviation); i.e., 0.5 h = 30 min.

**Table 4:** Full Sample Multilevel Models.

Predictors	ABC	Socialization	Communication	Daily Living Score
(Intercept)	0.10 (0.05) ***	0.08 (0.05) ***	−0.03 (0.05) ***	0.11 (0.05) ***
Time (mean-centered)	0.08 (0.02) ***	0.10 (0.02) *	0.04 (0.02) ^	0.11 (0.02) **
Cohort	−0.51 (0.10) ***	−0.36 (0.10) *	−0.20 (0.11) ^	−0.53 (0.10) ***
Age	−0.05 (0.03) ^	−0.15 (0.03) ***	−0.01 (0.01)	−0.05 (0.02) ^
Cohort × Time	−0.13 (0.02) ***	−0.07 (0.03) **	−0.12 (0.02) **	−0.13 (0.03) **

Note. Standardized beta (standard error); ^ n.s.; * *p* < 0.05; ** *p* < 0.01; *** *p* < 0.001. ABC: Adaptive Behavior Composite.

**Table 5:** Propensity-Weighted Multilevel Models.

Predictors	ABC	Socialization	Communication	Daily Living Score
(Intercept)	0.05 (0.05) ***	0.03 (0.05) ***	−0.02 (0.05) ***	0.06 (0.05) ***
Time (mean-centered)	0.08 (0.02) ***	0.04 (0.02) *	0.05 (0.02) ***	0.07 (0.02) ***
Cohort	−0.48 (0.10) ***	−0.24 (0.10) *	−0.09 (0.11) ^	−0.40 (0.10) ***
Age	−0.07 (0.03) ^	−0.15 (0.03) ***	−0.01 (0.01) ^	−0.03 (0.02) ^
Cohort × Time	−0.24 (0.02) ***	−0.22 (0.03) ***	−0.31 (0.02) ***	−0.22 (0.03) ***

Note. Standardized beta (standard error); ^ n.s.; * *p* < 0.05; *** *p* < 0.001. ABC: Adaptive Behavior Composite.

## Data Availability

The datasets used in the current study are available from the corresponding author on reasonable request. Due to the sensitive and privacy-protected nature of clinical data, access may be granted only under appropriate safeguards and ethical approvals.

## Supplementary Materials

The following supporting information can be downloaded at: https://www.mdpi.com/article/10.3390/jpm16050242/s1, Table S1: Probit Regression Model for Propensity Score Estimation; Table S2: Model fit at each step of the model-building sequence.
