# Supplementary material for: Adaptive Behavior Change in Autism: Outcomes from a Comprehensive, Interdisciplinary Clinical Care Cohort
Source: J Pers Med. 2026 Apr 30;16(5):242. doi: 10.3390/jpm16050242 (PMC13208269; doi:10.3390/jpm16050242)
Supplement: Supplementary file 1 [file jpm-16-00242-s001.zip › jpm-3900622-supplementary.pdf]

Supplementary Table S1. Probit Regression Model for Propensity Score Estimation

	**b**	**SE**	**z**	**p**
Intercept	-0.364	0.196	-1.86	.063
Baseline ABC	0.019	0.003	6.70	<.001

Note. Nagelkerke's R^2*=0.039. Cohort membership (0 = NDAR, 1 = Cortica) regressed on baseline VABS ABC Composite score using probit regression. VABS: Vineland Adaptive Behavior Scales; ABC: Adaptive Behavior Composite.*

Supplemental Table S2. Model fit at each step of the model-building sequence

**Outcome**	**Model**	**npar**	**AIC**	**BIC**	**χ²**	**Df**	**p**
ABC (Adaptive Behavior Composite)	Null	3	14582	14598	—	—	—
	Predictor	6	14548	14581	39.5	3	<.001
	Interaction	7	14489	14528	61.31	1	<.001
Socialization	Null	3	15494	15511	—	—	—
	Predictor	6	15459	15493	41.31	3	<.001
	Interaction	7	15432	15471	28.95	1	<.001
Communication	Null	3	15855	15871	—	—	—
	Predictor	6	15837	15871	23.44	3	<.001
	Interaction	7	15776	15816	62.69	1	<.001
Daily Living	Null	3	15248	15264	—	—	—
	Predictor	6	15225	15258	28.54	3	<.001
	Interaction	7	15195	15234	31.95	1	<.001
